# 4-(2,2-Difluoro-1,3-benzodioxol-4-yl)-1*H*-pyrrole-3-carbonitrile

**DOI:** 10.1107/S1600536811054523

**Published:** 2011-12-23

**Authors:** Fan-Wei Meng, Guang-Feng Hou, Ying-Hui Yu, Jin-Sheng Gao

**Affiliations:** a Engineering Research Center of Pesticides of Heilongjiang University, Heilongjiang University, Harbin 150050, People’s Republic of China, and College of Chemistry and Materials Science, Heilongjiang University, Harbin 150080, People’s Republic of China

## Abstract

In the title compound, C_12_H_6_F_2_N_2_O_2_, the 2,2-difluoro-1,3-benzodioxole ring system is approximately planar [maximum deviation = 0.012 (2) Å] and its mean plane is twisted with respect to the pyrrole ring, making a dihedral angle of 2.51 (9)°. In the crystal, N—H⋯N hydrogen bonds link the mol­ecules into chains running along the *a* axis. π–π stacking is also observed between parallel benzene rings of adjacent mol­ecules, the centroid–centroid distance being 3.7527 (13) Å.

## Related literature

For background to the title compound, see: Li *et al.* (2009 ▶); Pfluger *et al.* (1990 ▶). For the synthesis, see: Nyfeler & Ehrenfreund (1986 ▶). 
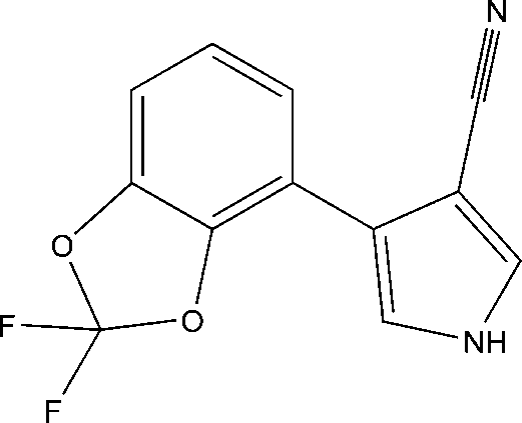


## Experimental

### 

#### Crystal data

C_12_H_6_F_2_N_2_O_2_
*M* *_r_* = 248.19
Triclinic, 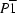

*a* = 7.5726 (15) Å
*b* = 7.8114 (16) Å
*c* = 8.9785 (18) Å
α = 93.58 (3)°
β = 94.65 (3)°
γ = 97.47 (3)°
*V* = 523.42 (18) Å^3^
*Z* = 2
Mo *K*α radiation
μ = 0.13 mm^−1^
*T* = 293 K
0.39 × 0.32 × 0.15 mm

#### Data collection

Rigaku R-AXIS RAPID diffractometer
Absorption correction: multi-scan (*ABSCOR*; Higashi, 1995 ▶) *T*_min_ = 0.950, *T*_max_ = 0.980
5120 measured reflections
2359 independent reflections
1485 reflections with *I* > 2σ(*I*)
*R*_int_ = 0.026

#### Refinement

*R*[*F* ^2^ > 2σ(*F* ^2^)] = 0.043
*wR*(*F* ^2^) = 0.118
*S* = 1.04
2359 reflections
167 parameters
1 restraint
H atoms treated by a mixture of independent and constrained refinement
Δρ_max_ = 0.19 e Å^−3^
Δρ_min_ = −0.15 e Å^−3^

### 

Data collection: *RAPID-AUTO* (Rigaku, 1998 ▶); cell refinement: *RAPID-AUTO*; data reduction: *CrystalClear* (Rigaku/MSC, 2002 ▶); program(s) used to solve structure: *SHELXTL* (Sheldrick, 2008 ▶); program(s) used to refine structure: *SHELXTL*; molecular graphics: *SHELXTL*; software used to prepare material for publication: *SHELXTL*.

## Supplementary Material

Crystal structure: contains datablock(s) I, global. DOI: 10.1107/S1600536811054523/xu5407sup1.cif

Structure factors: contains datablock(s) I. DOI: 10.1107/S1600536811054523/xu5407Isup2.hkl

Supplementary material file. DOI: 10.1107/S1600536811054523/xu5407Isup3.cml

Additional supplementary materials:  crystallographic information; 3D view; checkCIF report

## Figures and Tables

**Table 1:** Hydrogen-bond geometry (Å, °)

*D*—H⋯*A*	*D*—H	H⋯*A*	*D*⋯*A*	*D*—H⋯*A*
N1—H101⋯N2^i^	0.89 (1)	2.15 (1)	3.034 (2)	169 (2)

Symmetry code: (i) 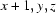.

**Fig. 1.**
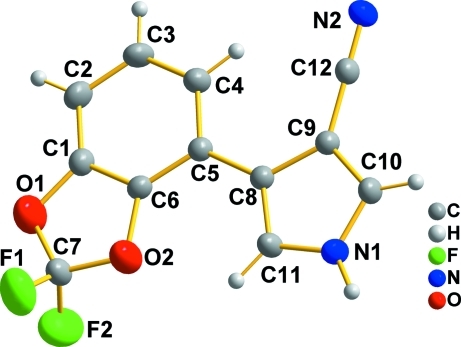
The molecular structure of the title compound, showing displacement ellipsoids at the 50% probability level for non-H atoms.

**Fig. 2.**
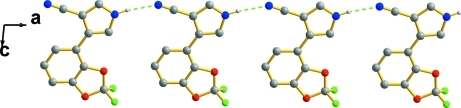
A partial packing view, showing the hydrogen-bonding chain structure along [100].

## supplementary crystallographic information

### Comment

Fludioxonil also know as Maxim, which is kind of fungicide developed and produced by Novartis (Li *et al.*, 2009; Pfluger *et al.*, 1990). Herein we report its structure.

In the title compound, phenyl and pyrrole ring are almost coplanar with a small dihedral angle of 2.51 (9)° (Figure 1). Intermolecular N—H···N hydrogen bonds link molecules into chains along [100] (Figure 2, Table 1).

### Experimental

The title compound was prepared by the reaction of 2-cyano-3-(2,2-difluoro-1,3-benzodioxol-4-yl)-2-propenamide and tosylmethyl isocyanide under alkaline condition (Robert & Josef, 1986). Colorless block crystals suitable for singl crystal X-ray diffraction were obtained by the recrystallization of title compound from a dichloromethane solution.

### Refinement

N-bound H atom was located in a differece Fourier map and positional parameters were refined, U_iso_(H) = 1.5U_eq_(N). Other H atoms were placed in calculated positions with C—H = 0.93 Å, and refined in riding mode with U_iso_(H) = 1.2U_eq_(C).

### Crystal data

**Table:**

C_12_H_6_F_2_N_2_O_2_	*Z* = 2
*M**_r_* = 248.19	*F*(000) = 252
Triclinic, *P*1	*D*_x_ = 1.575 Mg m^−^^3^
Hall symbol: -P 1	Mo *K*α radiation, λ = 0.71073 Å
*a* = 7.5726 (15) Å	Cell parameters from 3390 reflections
*b* = 7.8114 (16) Å	θ = 3.4–27.5°
*c* = 8.9785 (18) Å	µ = 0.13 mm^−^^1^
α = 93.58 (3)°	*T* = 293 K
β = 94.65 (3)°	Block, colorless
γ = 97.47 (3)°	0.39 × 0.32 × 0.15 mm
*V* = 523.42 (18) Å^3^	

### Data collection

**Table:**

Rigaku R-AXIS RAPID diffractometer	2359 independent reflections
Radiation source: fine-focus sealed tube	1485 reflections with *I* > 2σ(*I*)
graphite	*R*_int_ = 0.026
ω scan	θ_max_ = 27.5°, θ_min_ = 3.4°
Absorption correction: multi-scan (*ABSCOR*; Higashi, 1995)	*h* = −9→9
*T*_min_ = 0.950, *T*_max_ = 0.980	*k* = −10→10
5120 measured reflections	*l* = −11→10

### Refinement

**Table:**

Refinement on *F*^2^	Secondary atom site location: difference Fourier map
Least-squares matrix: full	Hydrogen site location: inferred from neighbouring sites
*R*[*F*^2^ > 2σ(*F*^2^)] = 0.043	H atoms treated by a mixture of independent and constrained refinement
*wR*(*F*^2^) = 0.118	*w* = 1/[σ^2^(*F*_o_^2^) + (0.0612*P*)^2^ + 0.0143*P*] where *P* = (*F*_o_^2^ + 2*F*_c_^2^)/3
*S* = 1.04	(Δ/σ)_max_ = 0.001
2359 reflections	Δρ_max_ = 0.19 e Å^−^^3^
167 parameters	Δρ_min_ = −0.15 e Å^−^^3^
1 restraint	Extinction correction: *SHELXTL* (Sheldrick, 2008), Fc^*^=kFc[1+0.001xFc^2^λ^3^/sin(2θ)]^-1/4^
Primary atom site location: structure-invariant direct methods	Extinction coefficient: 0.020 (6)

### Special details

**Table:**

Geometry. All esds (except the esd in the dihedral angle between two l.s. planes) are estimated using the full covariance matrix. The cell esds are taken into account individually in the estimation of esds in distances, angles and torsion angles; correlations between esds in cell parameters are only used when they are defined by crystal symmetry. An approximate (isotropic) treatment of cell esds is used for estimating esds involving l.s. planes.
Refinement. Refinement of F^2^ against ALL reflections. The weighted R-factor wR and goodness of fit S are based on F^2^, conventional R-factors R are based on F, with F set to zero for negative F^2^. The threshold expression of F^2^ > 2sigma(F^2^) is used only for calculating R-factors(gt) etc. and is not relevant to the choice of reflections for refinement. R-factors based on F^2^ are statistically about twice as large as those based on F, and R- factors based on ALL data will be even larger.

### Fractional atomic coordinates and isotropic or equivalent isotropic displacement parameters (Å^2^)

**Table:**

	*x*	*y*	*z*	*U*_iso_*/*U*_eq_	
C1	0.0680 (2)	0.6305 (2)	0.7663 (2)	0.0492 (4)	
C2	−0.1137 (2)	0.6131 (3)	0.7677 (2)	0.0580 (5)	
H2	−0.1723	0.5712	0.8478	0.070*	
C3	−0.2036 (2)	0.6626 (3)	0.6407 (2)	0.0604 (5)	
H3	−0.3277	0.6534	0.6348	0.072*	
C4	−0.1158 (2)	0.7254 (2)	0.5222 (2)	0.0533 (5)	
H4	−0.1833	0.7566	0.4395	0.064*	
C5	0.0715 (2)	0.7442 (2)	0.52139 (18)	0.0412 (4)	
C6	0.1551 (2)	0.6918 (2)	0.64884 (19)	0.0425 (4)	
C7	0.3564 (2)	0.6277 (3)	0.8215 (2)	0.0580 (5)	
C8	0.1679 (2)	0.8093 (2)	0.39662 (18)	0.0406 (4)	
C9	0.0963 (2)	0.8705 (2)	0.25982 (19)	0.0424 (4)	
C10	0.2365 (2)	0.9151 (2)	0.1754 (2)	0.0515 (5)	
H10	0.2281	0.9581	0.0812	0.062*	
C11	0.3488 (2)	0.8223 (3)	0.3852 (2)	0.0523 (5)	
H11	0.4323	0.7921	0.4572	0.063*	
C12	−0.0831 (2)	0.8924 (2)	0.2144 (2)	0.0474 (4)	
F1	0.46357 (16)	0.74161 (18)	0.91577 (13)	0.0804 (4)	
F2	0.43750 (16)	0.48669 (18)	0.81288 (16)	0.0803 (4)	
N1	0.38690 (19)	0.8860 (2)	0.25235 (18)	0.0575 (5)	
H101	0.4958 (17)	0.901 (3)	0.220 (3)	0.086*	
N2	−0.2273 (2)	0.9115 (2)	0.17807 (19)	0.0631 (5)	
O1	0.19296 (17)	0.5902 (2)	0.87638 (15)	0.0650 (4)	
O2	0.33825 (15)	0.69183 (17)	0.68299 (13)	0.0540 (4)	

### Atomic displacement parameters (Å^2^)

**Table:**

	*U*^11^	*U*^22^	*U*^33^	*U*^12^	*U*^13^	*U*^23^
C1	0.0499 (10)	0.0565 (11)	0.0440 (10)	0.0091 (8)	0.0105 (8)	0.0133 (8)
C2	0.0507 (10)	0.0722 (13)	0.0562 (12)	0.0089 (9)	0.0222 (9)	0.0210 (10)
C3	0.0379 (9)	0.0835 (14)	0.0652 (13)	0.0130 (9)	0.0160 (8)	0.0240 (11)
C4	0.0403 (9)	0.0718 (12)	0.0515 (11)	0.0115 (8)	0.0092 (8)	0.0185 (10)
C5	0.0381 (8)	0.0465 (9)	0.0405 (9)	0.0070 (7)	0.0077 (7)	0.0078 (7)
C6	0.0354 (8)	0.0514 (9)	0.0422 (9)	0.0057 (7)	0.0092 (7)	0.0078 (8)
C7	0.0467 (10)	0.0838 (14)	0.0474 (11)	0.0123 (10)	0.0066 (8)	0.0252 (10)
C8	0.0374 (8)	0.0470 (9)	0.0386 (9)	0.0068 (7)	0.0065 (7)	0.0072 (7)
C9	0.0382 (8)	0.0512 (10)	0.0396 (9)	0.0084 (7)	0.0061 (7)	0.0081 (7)
C10	0.0451 (9)	0.0727 (12)	0.0402 (10)	0.0117 (8)	0.0082 (7)	0.0193 (9)
C11	0.0388 (9)	0.0759 (12)	0.0461 (11)	0.0121 (8)	0.0066 (7)	0.0224 (9)
C12	0.0442 (10)	0.0602 (11)	0.0398 (10)	0.0085 (8)	0.0064 (7)	0.0134 (8)
F1	0.0666 (8)	0.1180 (11)	0.0512 (7)	−0.0057 (7)	−0.0045 (6)	0.0148 (7)
F2	0.0734 (8)	0.0941 (9)	0.0855 (10)	0.0318 (7)	0.0215 (6)	0.0415 (8)
N1	0.0377 (8)	0.0870 (12)	0.0525 (10)	0.0100 (8)	0.0134 (7)	0.0254 (8)
N2	0.0437 (9)	0.0914 (13)	0.0580 (11)	0.0144 (8)	0.0042 (7)	0.0246 (9)
O1	0.0525 (8)	0.1003 (11)	0.0473 (8)	0.0114 (7)	0.0113 (6)	0.0335 (7)
O2	0.0382 (6)	0.0824 (9)	0.0444 (7)	0.0082 (6)	0.0065 (5)	0.0242 (6)

### Geometric parameters (Å, °)

**Table:**

C1—C2	1.366 (3)	C7—F1	1.331 (2)
C1—C6	1.368 (2)	C7—O1	1.372 (2)
C1—O1	1.391 (2)	C7—O2	1.373 (2)
C2—C3	1.383 (3)	C8—C11	1.373 (2)
C2—H2	0.9300	C8—C9	1.437 (2)
C3—C4	1.382 (2)	C9—C10	1.375 (2)
C3—H3	0.9300	C9—C12	1.421 (2)
C4—C5	1.407 (2)	C10—N1	1.336 (2)
C4—H4	0.9300	C10—H10	0.9300
C5—C6	1.373 (2)	C11—N1	1.358 (2)
C5—C8	1.468 (2)	C11—H11	0.9300
C6—O2	1.3960 (19)	C12—N2	1.145 (2)
C7—F2	1.330 (2)	N1—H101	0.891 (10)
			
C2—C1—C6	122.93 (17)	F2—C7—O2	109.90 (18)
C2—C1—O1	127.94 (16)	F1—C7—O2	109.89 (16)
C6—C1—O1	109.12 (15)	O1—C7—O2	110.86 (15)
C1—C2—C3	114.73 (17)	C11—C8—C9	104.79 (14)
C1—C2—H2	122.6	C11—C8—C5	126.80 (15)
C3—C2—H2	122.6	C9—C8—C5	128.40 (14)
C4—C3—C2	122.42 (16)	C10—C9—C12	123.08 (16)
C4—C3—H3	118.8	C10—C9—C8	107.74 (15)
C2—C3—H3	118.8	C12—C9—C8	129.12 (15)
C3—C4—C5	122.74 (17)	N1—C10—C9	108.11 (15)
C3—C4—H4	118.6	N1—C10—H10	125.9
C5—C4—H4	118.6	C9—C10—H10	125.9
C6—C5—C4	112.87 (15)	N1—C11—C8	109.45 (15)
C6—C5—C8	123.29 (14)	N1—C11—H11	125.3
C4—C5—C8	123.83 (15)	C8—C11—H11	125.3
C1—C6—C5	124.29 (15)	N2—C12—C9	179.4 (2)
C1—C6—O2	108.24 (15)	C10—N1—C11	109.90 (14)
C5—C6—O2	127.46 (14)	C10—N1—H101	125.5 (15)
F2—C7—F1	105.63 (16)	C11—N1—H101	124.5 (15)
F2—C7—O1	110.18 (16)	C7—O1—C1	105.74 (14)
F1—C7—O1	110.26 (18)	C7—O2—C6	106.02 (13)

### Hydrogen-bond geometry (Å, °)

**Table:**

*D*—H···*A*	*D*—H	H···*A*	*D*···*A*	*D*—H···*A*
N1—H101···N2^i^	0.89 (1)	2.15 (1)	3.034 (2)	169 (2)

Symmetry codes: (i) *x*+1, *y*, *z*.
